# Rapid expression of transgenes driven by seed-specific constructs in leaf tissue: DHA production

**DOI:** 10.1186/1746-4811-6-8

**Published:** 2010-03-11

**Authors:** James R Petrie, Pushkar Shrestha, Qing Liu, Maged P Mansour, Craig C Wood, Xue-Rong Zhou, Peter D Nichols, Allan G Green, Surinder P Singh

**Affiliations:** 1 CSIRO Food Futures National Research Flagship, PO Box 1600, Canberra, ACT 2601, Australia; 2 CSIRO Plant Industry, PO Box 1600, Canberra, ACT 2601, Australia

## Abstract

**Background:**

Metabolic engineering of seed biosynthetic pathways to diversify and improve crop product quality is a highly active research area. The validation of genes driven by seed-specific promoters is time-consuming since the transformed plants must be grown to maturity before the gene function can be analysed.

**Results:**

In this study we demonstrate that genes driven by seed-specific promoters contained within complex constructs can be transiently-expressed in the *Nicotiana benthamiana *leaf-assay system by co-infiltrating the *Arabidopsis thaliana *LEAFY COTYLEDON2 (LEC2) gene. A real-world case study is described in which we first assembled an efficient transgenic DHA synthesis pathway using a traditional *N. benthamiana *Cauliflower Mosaic Virus (CaMV) 35S-driven leaf assay before using the LEC2-extended assay to rapidly validate a complex seed-specific construct containing the same genes before stable transformation in *Arabidopsis*.

**Conclusions:**

The LEC2-extended *N. benthamiana *assay allows the transient activation of seed-specific promoters in leaf tissue. In this study we have used the assay as a rapid preliminary screen of a complex seed-specific transgenic construct prior to stable transformation, a feature that will become increasingly useful as genetic engineering moves from the manipulation of single genes to the engineering of complex pathways. We propose that the assay will prove useful for other applications wherein rapid expression of transgenes driven by seed-specific constructs in leaf tissue are sought.

## Background

**Figure 1:**
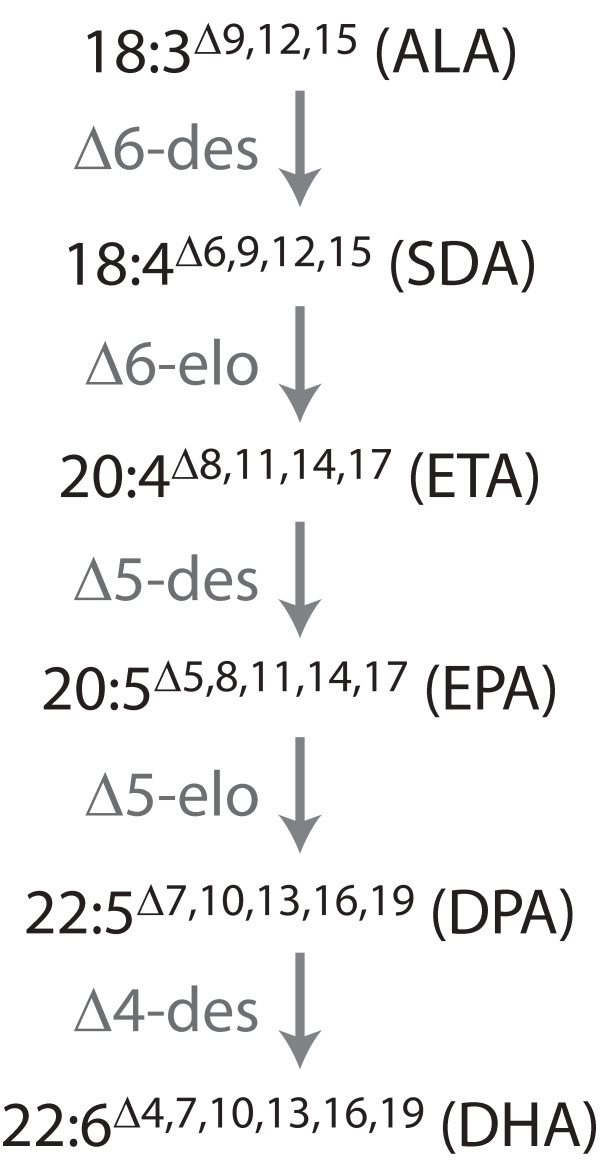
**DHA synthesis pathway**. The omega-3 long chain polyunsaturated fatty acid synthesis pathway described in this study. 'Des' refers to desaturase and 'elo' refers to elongase. The fatty acids are ALA, α-linolenic acid; SDA, stearidonic acid; ETA, eicosatetraenoic acid; EPA, eicosapentaenoic acid; DPA, docosapentaenoic acid; DHA, docosahexaenoic acid.

Metabolic engineering of seed biosynthetic pathways to diversify and improve crop product quality is a highly active research area. However, the time and resources required for this research are considerable, in part due to the complexity of the modifications sought. Crop plants carrying transgenes driven by seed-specific promoters must be grown to maturity, and in many cases through to the second generation, before the resulting trait can be adequately assessed. The engineering of long-chain (≥ C20) polyunsaturated fatty acids (LC-PUFA), and in particular EPA (eicosapentaenoic acid, 20:5^Δ5,8,11,14,17^) and DHA (docosahexaenoic acid, 22:6^Δ4,7,10,13,16,19^), into oilseed crop plants is a powerful example, with DHA biosynthesis typically requiring the introduction of a minimum of five novel, seed-specific, enzymatic steps (Fig. 1). Several studies have indicated that deficiencies in these fatty acids increase the risk of cardiovascular diseases, inflammatory diseases and depression and since humans are generally unable to synthesise adequate amounts of DHA we must obtain this fatty acid through our diet [1]. Many countries, however, have average daily intakes of EPA and DHA well below the recommended amounts and concern about the sustainability of wild fisheries and their ability to meet increasing demand has stimulated research into the production of land-based sources of these oils [2-4].

The P19 silencing-suppressed *N. benthamiana *assay has previously been used to transiently-express an entire functional DHA biosynthesis pathway in leaf tissue by mixing and co-infiltrating *Agrobacterium *strains harbouring single-gene 35S-driven constructs [5]. Although successful in identifying gene combinations capable of directing DHA synthesis, this approach could not be used to validate corresponding constructs destined for use in oilseeds since the genes in these constructs would be driven by seed-specific promoters that are not active in *N. benthamiana *leaf tissue. It is worth noting that seed-specific promoters are often used in a seed-trait engineering context both to obtain good expression and to limit the modified metabolites to the seed. This is especially important when engineering unusual oil pathways since the presence of these fatty acids in vegetative tissue can be deleterious to membrane structure and function.

Precise metabolic engineering of complex pathways requires the optimisation of multiple steps - a challenging prospect when limited to stable transformation and analysis of seed-expressed traits. We were therefore interested in modifying the *N. benthamiana *assay to allow the activation of seed-specific promoters in the leaf tissue. We hypothesised that extending the *N. benthamiana *leaf assay with the *A. thaliana *LEC2 transcription factor might allow rapid functional assessment of a complex construct designed for seed-specific expression in a land plant. The LEC2 transcription factor is currently the subject of considerable research due to its high-level control of entire metabolic pathways and LEC2 expression is known to establish a cellular environment that promotes the broad metabolic changes involved in embryo development [6,7].

In this study we demonstrate the use of a LEC2-extended *N. benthamiana *transient assay in which the leaf expression of five genes driven by seed-specific promoters contained within a single construct resulted in the synthesis of the omega-3 polyunsaturated fatty acid DHA. The ability to rapidly express complex seed-specific constructs in leaf tissue will be increasingly useful as genetic engineering moves from the manipulation of single genes to the engineering of complex pathways.

## Results and Discussion

### Identification of an efficient DHA synthesis pathway

We first used the original *N. benthamiana *leaf assay (without LEC2 co-infiltration) to rapidly identify a combination of genes that resulted in efficient DHA synthesis. We transiently-expressed an entire DHA pathway containing the Δ6-desaturase from *Micromonas pusilla *to convert ALA to stearidonic acid (SDA, 18:4^Δ6,9,12,15^), the *Pyramimonas cordata *Δ6-elongase for conversion of SDA to eicosatetraenoic acid (ETA, 20:4^Δ8,11,14,17^) and the highly efficient *P. salina *Δ5-desaturase for the production of EPA from ETA [8,9]. The *P. cordata *Δ5-elongase was then used to convert EPA to docosapentaenoic acid (DPA, 22:5^Δ7,10,13,16,19^) before the final step in the pathway (conversion to DHA) was catalysed by the *P. salina *Δ4-desaturase [8-10]. The genes were combined in *N. benthamiana *by infiltrating in triplicate a mixture of *Agrobacterium *strains, each carrying a single construct, into the abaxial surface of the leaf in the presence of the P19 viral suppressor. Fatty acid methyl esters (FAME) were produced from this leaf tissue five days after agroinfiltration and analysed by GC (Table 1).

**Table 1:** *Nicotiana benthamiana *leaf fatty acid profiles

Fatty acid			Construct pJP3057	
**Usual FA**	**P19 only**	**35S DHA mix**	- **35S:LEC2**	**+ 35S:LEC2**

16:0	15.9 ± 0.2	16.6 ± 0.1	13.3 ± 0.1	13.2 ± 0.6
16:1^Δ3t^	1.7 ± 0.1	1.5 ± 0.1	1.3 ± 0.1	1.1 ± 0
16:3^Δ9,12,15^	6.3 ± 0.3	5.6 ± 0.1	7.1 ± 0.3	7.5 ± 0.4
18:0	3.6 ± 0.3	3.3 ± 0.1	1.8 ± 0.1	2.4 ± 0.3
18:1^Δ9^	2.8 ± 0.1	2.8 ± 0.2	1.1 ± 0.1	1.5 ± 0.2
18:2^Δ9,12^	18.7 ± 0.1	13.0 ± 0.1	13.8 ± 0.1	12.7 ± 0.4
18:3^Δ9,12,15^	45.6 ± 1.4	40.2 ± 0.5	56.3 ± 0.7	44.8 ± 2.1
20:0	1.3 ± 0.4	0.6 ± 0	0.3 ± 0	0.5 ± 0.1
Other minor	4.1	6.6	3.9	4.8

Total	100	90.4	98.9	88.5

				
New ω6 PUFA				

18:3^Δ6,9,12^	-	2.1 ± 0.2	-	2.4 ± 0.1
20:3^Δ8,11,14^	-	-	0.2 ± 0.1	0.2 ± 0.1
20:4^Δ5,8,11,14^	-	0.2 ± 0	-	-

Total	0	2.3	0.2	2.6

				
New ω3 PUFA				

18:4^Δ6,9,12,15^	-	2.0 ± 0 (16% Δ6-des)	0.9 ± 0.1	1.2 ± 0.1 (15% Δ6-des)
20:4^Δ8,11,14,17^	-	0.4 ± 0 (75% Δ6-elo)	-	2.0 ± 0.1 (85% Δ6-elo)
**20:5Δ^5,8,11,14,17^**	-	0.3 ± 0 (93% Δ5-des)	-	0.6 ± 0 (71% Δ5-des)
22:5^Δ7,10,13,16,19^	-	2.3 ± 0 (94% Δ5-elo)	-	1.7 ± 0.1 (88% Δ5-elo)
**22:6Δ^4,7,10,13,16,19^**	-	2.5 ± 0.1 (52% Δ4-des)	-	2.5 ± 0.2 (60% Δ4-des)

Total	0	7.5	0.9	11.1

				
Total new FA	0	14.0	1.1	13.0

Total FA	100	100	100	100

Profile of fatty acid methyl esters produced directly from *N. benthamiana *leaf tissue transiently expressing single-gene CaMV 35S binary constructs and the seed-specific construct pJP3057. All infiltrations were performed with the P19 gene silencing suppressor and errors denote standard deviation of separate infiltrations performed in triplicate. Where shown, conversion efficiencies are calculated as *p*/(*s *+ *p*) where *p *and *s *are the sum of all downstream products or substrates, respectively, and where both *p *and *s *are expressed as molar percentage of total fatty acids.

All genes were found to be active and DHA was produced by the transgenic pathway. Use of the highly efficient *P. cordata *Δ5-elongase resulted in very low EPA accumulation in total leaf lipids (0.3%) with 94% being elongated to DPA. Importantly, this pathway resulted in the synthesis of very low levels of intermediate ω3 fatty acids due to the high conversion efficiencies achieved by the enzymes. There was also an almost complete absence of ω6 fatty acids due to the ω3-preference displayed by the *M. pusilla *Δ6-desaturase. This *N. benthamiana *leaf assay was useful in identifying a combination of genes that resulted in efficient DHA production but was seriously limited by requiring the use of independent, 35S-driven, genes. A pathway in this configuration is not useful in a stable seed context so we next attempted to extend the leaf assay to allow the analysis of such complex, seed-specific, constructs.

### LEC2-enabled transient-expression of seed-specific promoters in leaf

**Figure 2:**
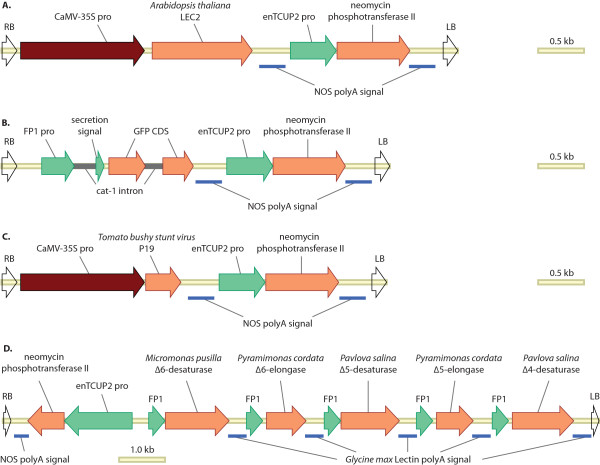
**Construct maps**. Maps of the intra-border regions of the binary vectors described in this study. **A**. is the 35S:LEC2 construct; **B**. is the FP1:GFP construct; **C**. is the 35S:P19 construct included in all infiltrations; **D**. DHA synthesis construct pJP3057 with each gene driven by FP1, the truncated napin promoter from *Brassica napus*.

**Figure 3:**
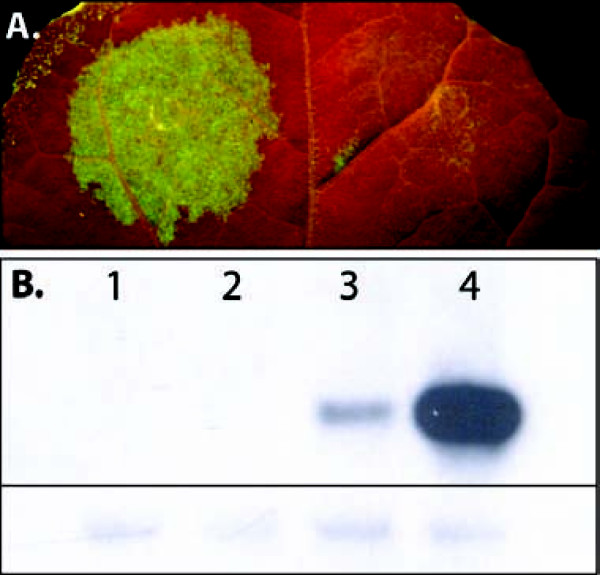
**Typical seed storage protein promoter expression in leaf**. **A**. *Nicotiana benthamiana *leaf transformed with *Agrobacterium *strains carrying the individual constructs 35S:P19, FP1:GFP and 35S:LEC2 (left side of leaf only) with the seed-specific FP1-driven GFP observed only in the presence of 35S:LEC2. **B**. Western blot of *N. benthamiana *leaf assays, upper panel showing specific bands for GFP at 30 kDa and lower panel showing non-specific background bands from the same exposure indicative of equal loading between samples. Lane 1: wildtype leaf; Lane 2: 35S:P19 + 35S:LEC2; Lane 3: 35S:P19 + FP1:GFP (Fig. 3A, right); Lane 4: 35S:P19 + 35S:LEC2 + FP1:GFP (Fig. 3A, left).

Stone et al. [7] reported on the strong embryogenic effects of LEC2 expression and we postulated that even transient-expression of LEC2 may allow leaf expression of promoters that would usually be active only in a seed. We first tested whether a promoter from a typical *Brassica napus *seed storage protein, napin, would be activated in *N. benthamiana *leaf tissue when co-infiltrated with LEC2. The construct 35S:LEC2 (Fig. 2A) was built by cloning the *A. thaliana *LEC2 gene into a binary vector between a CaMV-35S promoter and the *A. tumefaciens *NOS polyadenylation signal. A second construct, FP1:GFP (Fig. 2B), was made by cloning an intron-interrupted, secreted GFP gene into a binary vector between a truncated *Brassica napus *napin promoter (FP1) and the NOS polyadenylation signal. Co-infiltration of the *A. tumefaciens *(AGL1) strains harbouring FP1:GFP and 35S:LEC2 along with 35S:P19 (Fig. 2C) resulted in strong GFP expression (Fig. 3A, left side). In contrast, infiltration of FP1:GFP and 35S:P19 alone resulted in minimal GFP expression (Fig. 3A, right side). Western blot confirmed that GFP expression was markedly increased in the presence of LEC2 (Fig. 3B). This conceptual demonstration of the capability of the LEC2-extended leaf assay to functionally activate a transiently-expressed seed-specific promoter encouraged us to experiment with more complex constructs.

### Rapid validation of a complex seed-specific construct in leaf

The five genes comprising the efficient DHA synthesis pathway identified above were built into the complex seed-specific construct pJP3057 (Fig. 2D) which was then validated in the LEC2-extended *N. benthamiana *leaf assay. Production of DHA was observed when pJP3057 was co-infiltrated with 35S:LEC2 and 35S:P19 whereas the infiltration of 35S:P19 and either pJP3057 or 35S:LEC2 alone did not result in any LC-PUFA production (Table 1). Whilst these results were no guarantee of the success of the construct in a stably-transformed event, they did indicate that there was nothing fundamentally unstable in the structure of the construct.

### Stable *Arabidopsis *transformation

The construct pJP3057 was then stably-transformed in *A. thaliana *(ecotype Columbia). Following transformation with pJP3057 and selection on kanamycin, 21 plants were recovered and the fatty acid profile of their seed analysed. The T_2 _seed from a representative T_1 _plants showed synthesis of DHA with amounts ranging from 0.2% to 2.4%. The highest DHA-containing line (JB-7, Table 2) was confirmed to be a single locus transformation event by the 3:1 segregation of the kanamycin-resistance gene in its T_2 _seed. It is interesting to note that the activity displayed by the *P. cordata *Δ5-elongase in both *N. benthamiana *and *A. thaliana *is by far the most efficient Δ5-elongation thus far reported *in planta *and use of this gene effectively overcomes the large Δ5-elongation bottleneck that has been experienced in other attempts at transgenic DHA production [11-14].

**Table 2:** Transgenic *Arabidopsis *seed fatty acid profiles

Fatty acid	Columbia	pJP3057 JB-7
Usual FA		
16:0	7.7	7.6
18:0	3.1	3.7
20:0	2.1	1.8
18:1^Δ9^	12.9	12.8
20:1^Δ11^	18.3	16.3
18:2^Δ9,12^	27.8	27.2
18:3^Δ9,12,15^	19.7	17.3
Other minor	8.4	8.0

Total	100	94.7

		
New ω6 PUFA		

18:3^Δ6,9,12^	-	1.4
20:3^Δ8,11,14^	-	-
20:4^Δ5,8,11,14^	-	-

Total	0	1.4

		
New ω3 PUFA		

18:4^Δ6,9,12,15^	-	0.4 (18% Δ6-des)
20:4^Δ8,11,14,17^	-	0.6 (90% Δ6-elo)
**20:5^Δ5,8,11,14,17^**	-	**0.2 (83% Δ5-des)**
22:5^Δ7,10,13,16,19^	-	0.3 (93% Δ5-elo)
**22:6^Δ4,7,10,13,16,19^**	-	**2.4 (89% Δ4-des)**

Total	0	3.9

		
Total new FA	0	5.3

Total FA	100	100

Profiles of fatty acid methyl esters produced directly from transgenic *Arabidopsis thaliana *(ecotype Columbia) seed stably transformed with construct pJP3057. Conversion efficiencies are calculated as in Table 1.

## Conclusions

We have demonstrated that it is possible to transiently-express genes driven by seed-specific promoters in leaf tissue to yield a functional metabolic pathway. The profound seed-like metabolic changes caused by the LEC2 transcription factor make the LEC2-extended system a more relevant background in which to assay genes and constructs destined for stable seed transformation than a traditional *N. benthamiana *leaf assay [7]. It is worth noting that further work is required to define the extent to which this assay could be useful in predicting construct function in a stably-transformed context. It will also be interesting to determine which seed-specific promoters can be similarly activated by LEC2 in leaf tissue. We expect this new assay will have multiple applications, not least of which will be to provide a 'rapid-fail' test for poorly designed seed-specific constructs that would traditionally take at least one plant generation to assess. As genetic engineering moves from the manipulation of single genes to engineering of complex pathways, an assay system such as described here will be invaluable in accelerating the rate of optimisation of engineered pathways.

## Methods

### Binary vector construction

Each of the individual 35S constructs was built by inserting an *Eco*RI-flanked gene coding region into the same site of a pORE04 binary vector with already contained the *A. tumefaciens *NOS polyadenylation signal and modified by the addition of a double CaMV-35S promoter at the *Sfo*I site [15]. The binary vector pJP3057 was built by first cloning the same *Eco*RI-flanked gene coding regions into the same site in an intermediate cloning vector between a truncated *Brassica napus *napin promoter, FP1, and the *A. tumefaciens *NOS polyadenylation signal. The entire cassette was then cloned into suitably adapted sites of the multiple cloning site in pORE04. 35S:P19 and the intron-interrupted, secreted GFP gene used in FP1:GFP was provided by Dr Peter Waterhouse.

### *N. benthamiana *leaf infiltration

Each *Agrobacterium tumefaciens *strain AGL1 harbouring a binary vector was grown at 28°C with shaking in LB broth supplemented with the appropriate antibiotics for two days. The amount of culture required to yield 1 mL of OD_600 nm _= 2.5 culture was centrifuged (10,000 g, 1 minute). After removal of the supernatant the pellet was gently resuspended in 1 mL of infiltration buffer (5 mM MES, 5 mM MgSO_4_, pH 5.7, 100 μM acetosyringone freshly added) and the culture incubated at 28°C with shaking for a further three hours. Each culture was then used as a 10× stock for culture mixture, with the remainder of the required volume made up by infiltration buffer. All *N. benthamiana *infiltrations included a 35S:P19 culture. The culture mixtures were infiltrated as described by Voinnet et al. [16], into the underside of leaves of approximately one month old *N. benthamiana *plants that had been housed in a 23°C plant growth room with 10:14 light:dark cycle but moved to 28°C with water two hours prior to infiltration. Following infiltration the infiltrated regions were circled with a permanent marker and the plants were left at 28°C for one hour after which they were transferred to a 24°C plant growth room for five days before being harvested using a leaf disc cutter.

### *A. thaliana *transformation

*A. thaliana *(ecotype Columbia) was used for plant transformations. *Agrobacterium*-mediated transformation was performed by the floral dipping method [17]. T_1 _seeds were harvested and plated on media containing 20 mg L^-1 ^kanamycin to test segregation ratios.

### Lipid analysis

Total lipid extraction, lipid class analysis, fatty acid methyl ester preparation and all analyses were performed as previously described [5].

## Competing interests

The authors declare that they have no competing interests.

## Authors' contributions

JRP participated in the design of the study, cloned constructs, performed benth infiltrations, analysed results and drafted the manuscript. PS, MPM and PDN performed lipid biochemistry and analysis work and contributed to the manuscript. QL, CCW and XZ contributed to the manuscript and gene cloning, with CCW building the FP1:GFP construct. AGG participated in the study design and contributed to the manuscript. SPS conceived of the LEC2 concept, participated in the design of the study, analysed results and contributed to the manuscript. All authors read and approved the final manuscript.
